# Biochemical and transcriptomic analyses of the symbiotic interaction between *Cremastra appendiculata* and the mycorrhizal fungus *Coprinellus disseminatus*

**DOI:** 10.1186/s12870-021-03388-6

**Published:** 2022-01-04

**Authors:** Yanyan Gao, Jun Ji, Yujin Zhang, Ningxian Yang, Mingsheng Zhang

**Affiliations:** 1 College of Life Sciences, Guizhou University, Guiyang, 550025 Guizhou China; 2 Key Laboratory of Plant Resources Conservation and Germplasm Innovation in Mountainous Region (Ministry of Education), Guiyang, 550025 Guizhou China

**Keywords:** *Cremastra appendiculata*, *Coprinellus disseminatus*, Lignin degradation, Transcriptome analysis, Seed germination

## Abstract

**Background:**

*Cremastra appendiculata* is a rare terrestrial orchid with a high market value as an ornamental and medicinal plant. However, the species depends entirely on fungi for seed germination under natural conditions. In a previous study, we have successfully isolated and identified the mycorrhizal fungus *Coprinellus disseminatus* which was able to induce the germination of *C. appendiculata* seeds. We then speculated that *C. disseminatus* may do so by breaking the testa imposed dormancy of the seeds. In this study, biochemical and transcriptomic analyses were used to characterize the germination of *C. appendiculata* seeds, collected at different stages of germination, as affected by *C. disseminatus*.

**Results:**

The lignocellulose in the seeds coat of *C. appendiculata* was degraded by the mycorrhizal fungus resulting in facilitated absorption of water. The rate of decline in lignin content was 67 and 73% at 6 and 12 days after sowing, respectively. The water content increased from 13 to 90% during symbiosis. A total of 15,382 genes showing significantly different levels of expression (log_2_ FPKM≥2.0, Qvalue≤0.05) were successfully identified among all libraries, where the highest number of DEGs was shared between 6 days versus 0 day after symbiotic germination. Gene annotation results suggested that 15 key genes related water-status, such as *DHN* gene family and *Xero* 1 were down-regulated. The genes zeaxanthin epoxidase *ZEP*, 9-cis-epoxycarotenoid dioxygenase *NCED3* and β-carotene hydroxylase involved in the biosynthesis of abscisic acid (ABA) were significantly down-regulated in 6 days as compared to 0 day after symbiotic germination.

**Conclusions:**

This work demonstrates that mycorrhizal fungus *C. disseminatus* can stimulate *C. appendiculata* seeds germination through a mechanism of breaking the testa imposed dormancy and inducing water absorption of the embryo.

**Supplementary Information:**

The online version contains supplementary material available at 10.1186/s12870-021-03388-6.

## Background

Orchidaceae is the most species-rich family in the plant kingdom with more than 28,000 known species [9]. Many orchid seeds are tiny dust-like, with lignified seed coats, which mainly depend on compatible mycobionts for germination and subsequent growth and survival of the seedlings [3, 6, 12, 18, 34, 45]. The lignified seed coats of the orchids arise mainly from the outer and inner integuments, and contain lignocellulose, suberin, polyphenols and cutin [4, 20, 34]. Lignin is a component of plant cell wall that contains hemicellulose, cellulose and lignin. Lignin binds to cellulose and hemicellulose forming a hydrophobic barrier to water permeation [27, 32]. This barrier enhances the survival of orchid seeds in harsh conditions and enhances wind-aided seed dispersal [4, 12]. However, the lignified seed coat may interfere with water and nutrient uptake, resulting in ‘physically imposed dormancy’ [4, 5, 12, 31, 34, 44]. Thus, effectively breaking the testa-imposed dormancy is a critical step in seed germination which a prerequisite for species conservation and large-scale production of orchids.

Orchid mycorrhizal fungi have been reported to be capable of degrading lignin by producing lignin-modifying enzymes and energetically supporting the establishment of orchid seedlings [34, 36, 40]. However, some studies have reported that an efficient enzymatic machinery of mycorrhizal fungi toward lignin was not sufficient for effective break down of lignocellulose in the seed coat, and that some fungi can produce appressorium-like structures or utilize conidia to soften the cell walls [11]. Moreover, the mycorrhizal fungi produce cellulases and pectinases, which contribute to effective penetration of the fungal hyphae into plant tissues [35]. White-rot fungi are recognized as one of the most active microorganisms in lignin degradation. The saprobic fungus *Coprinellus disseminatus* has been documented to be able to degrade lignocellulose [2, 39].

The lignified seed coat, which is thought to be one of the factors in seed dormancy, is formed during the final stages of seed development. Concomitantly, the highly hydrophilic dehydrin (*DHN*) proteins accumulate in the embryo and serve as protectants against desiccation [7, 10, 16, 28]. Dehydrins have multifaceted roles in the protection of plant cells during stress conditions, including drought which triggers ROS accumulation and negatively affects the overall plant performance [37, 48]. Phytohormones strongly regulate seed germination and stress responses. Among which ABA is a primary stress hormone that induces seed dormancy, and regulates stomatal closure to restrict water loss by transpiration [13, 24, 49]. The expression of *DHN* proteins has also been reported to be induced by both ABA-dependent or ABA-independent mechanisms under drought stress conditions [37].

*Cremastra appendiculata*, is a rare terrestrial orchid that often grows in the understory of humid and highly shaded forests [45]. It has a high medicinal value because its tubers are effective for cancer treatment, angiogenesis inhibition, heat-clearing, detoxification and detumescence [22, 23, 38, 41]. Due to its commercial and medicinal market value, *C. appendiculata* plant material often illegally collected from the wild populations. In a previous study, we reported that seeds of *C. appendiculata* with intense phenolic compounds in the coats showed enhanced germination and protocorm development in the presence of the mycorrhizal fungus *C. disseminatus* DJF-10 [14]. However, the mechanisms involved in the symbiotic germination remained unknown.

In this study, seeds at 0 (fungus unpenetrated), 6 (fungus penetrating), 12 (fungus penetrated into embryo and formed pelotons) and 25 days (seeds developed into protocorms) after symbiotic germination were used as test materials to investigate the influence of *C. disseminatus* on the lignocellulose of the seed. We studied the dynamic changes in the lignocellulose content of *C. appendiculata* seeds during symbiotic germination with *C. disseminatus* and then, analyzed the lignocellulose degradation by a combination of methods, including Fourier transform infrared spectroscopy (FTIR), pyrolysis-gas chromatography mass spectrometry (Py-GC/MS) and gas chromatography mass spectrometry (GC-MS). Finally, we analyzed transcriptome of *C. appendiculata* during symbiotic germination, which indicated that *C. disseminatus* possibly degrades lignin in the seed coat, with concomitant increase in water uptake.

## Materials and methods

### Experimental materials and symbiotic germination

The mature seeds of *C. appendiculata* and the mycorrhizal fungus *C. disseminatus* DJF-10 were obtained and stored in the Institute of Plant Physiology and Molecular Biology of Guizhou University, Guiyang city, Guizhou Province, China. The seeds were sterilized with 75% ethanol (30 s) and then with sodium hypochlorite (1% effective chloride, 3 min). The seeds were germinated in Petri dishes containing oatmeal agar medium (OMA: 4 g L^− 1^ rolled oats, 8 g L^− 1^ agar, at natural pH). Then a 5 mm-plug of actively growing *C. disseminatus* mycelium, pre-cultured on PDA medium, was placed in each OMA Petri dish. The plates were incubated at 22 ± 2 °C in full darkness.

### Phenotypes of *C. appendiculata* seeds during symbiotic germination with *C. disseminatus*

**Fig. 1:**
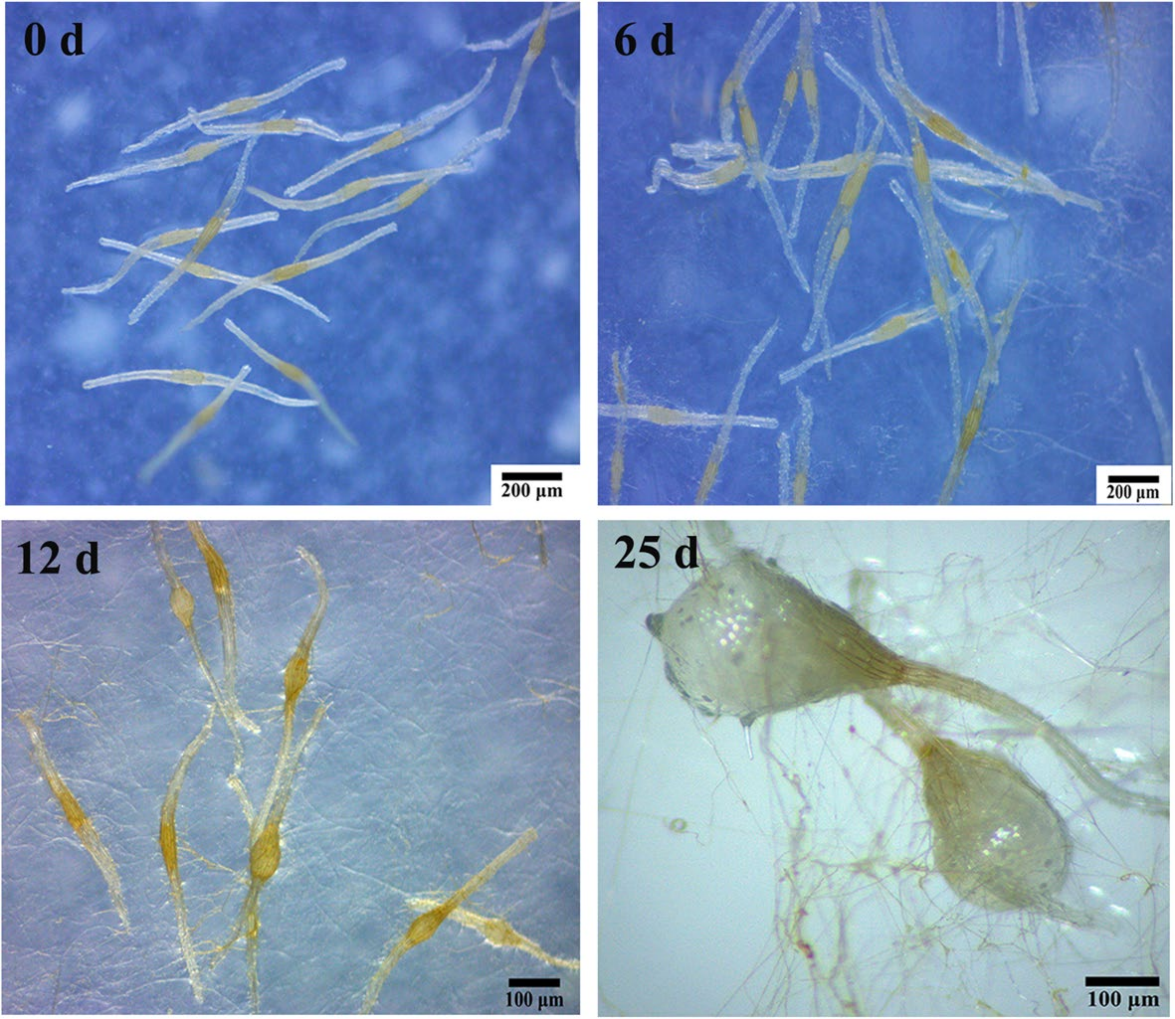
Phenotypes of the seeds of *C. appendiculata* symbiotically germinated with *C. disseminatus* DJF-10 at different stage after sowing. Zero day, six days, twelve days, twenty-five days indicated symbiotic seeds at 0 day, 6 days, 12 days and 25 days after sowing, respectively

The symbiotically germinated seeds were samples at four stages according to a previous histological study [14]. First, at symbiotic sowing (0 day) (before fungus invasion) where the embryo was surrounded with lignified seed coat. Second, at 6 days (fungus penetrating) where the mycelium penetrated from the suspensor. Third, at 12 days (fungus colonization) where some well-developed pelotons formed and the swollen embryo broke through the testa. Fourth at 25 days (pelotons degradation) where more than 70% of the seeds germinated and formed protocorms with protomeristem at the basal cells were degraded. Therefore, samples were collected at 0 (Fig. 1 0 day), 6 (Fig. 1 6 days), 12 (Fig. 1 12 days) and 25 days (Fig. 1 25 days) after symbiotic sowing (CA, SY1, SY2, and SY3, respectively) and stored at − 80 °C for transcriptome research and other subsequent analyses.

### Total lignocellulose content and water uptake in *C. appendiculata* germinating seeds

#### Lignocellulose content

The dried samples were milled using a pestle and stored in sealed plastic bags. The lignocellulose contents were examined using lignin content assay kit (Solarbio, China). The hemicellulose and cellulose contents were measured using respective assay kits (Sangon Biotech, China) according to manufacturer’s instructions.

#### Water uptake by seeds at different stages of germination

The symbiotic seeds at different stages of germination were dried at 40 °C until constant dry weight was reached (48–72 h). The water content was expressed as a percentage of the seed fresh weight for four to five biological replicates.

### FTIR and Py-GC/MS analysis of lignocellulose degradation

The FTIR was used to follow the degradation of lignocellulose with a Vertex70 (Bruker, Germany) FTIR spectrometer. Before the FTIR, the dried samples were embedded in KBr pellets at room temperature. FTIR spectrophotometer were recorded in a range of 4000 cm^− 1^ to 400 cm^− 1^. The spectra were recorded in an absorption mode of 16 scans per sample with a resolution of 2 cm^− 1^ [12, 46].

The chemical composition of lignocellulose from symbiotic seeds was analyzed by Py-GC/MS according the method of Chen et al. [8] with some modifications. Helium was used as the carrier gas at a constant rate of 1 mL min^− 1^. The pyrolysis was carried out at 550 °C, the chromatograph was programmed from 50 to 250 °C at a rate of 15 °C min^− 1^ and the final temperature was maintained for 10 min. The chemical compounds were identified by comparing their mass spectra with those at the National Institute of Standards and Technology (NIST) library.

### GC-MS analysis of low-molecular-weight degradation products of lignocellulose during symbiotic germination

To evaluate the lignocellulose degradation efficiency of the mycorrhizal fungus *C. disseminatus*, products from lignocellulose of *C. appendiculata* seeds were analyzed by GC-MS, since this system is appropriate for analysis of the low-molecular-weight compounds. The compounds were harvested by crushing the symbiotic medium with a rod and then stirring on shaker at 120 rpm at 10 °C for 4 h [2]. After filtering the contents through four layers of cheese cloth, the samples centrifuged at 10,000 rpm for 10 min at 4 °C. The supernatant was harvested and stored at − 20 °C. The pre-treatment of the supernatant was performed according to Zang et al. [46] with some modifications. The supernatants were acidified with concentrated HCl to pH 2–3. Then, the samples were extracted three times with ethyl acetate and the organic layers were combined. The combined fractions were dewatered over anhydrous Na_2_SO_4_ passed through a filter paper and evaporated down to 2 mL at 40 °C. Consequently, 100 μL of pyridine and 400 μL of N, O-bis (trimethylsilyl) trifluoroacetamide (BSTFA) were added to 500 μL samples of the extracted products. The samples were silylated with heating at 60 °C for 30 min and filtered through 0.45 μm membranes. Aliquots of 1 μL of the silylated samples were injected into the GS-MS system. Three replicates were used for each sample.

The GC-MS analysis achieved using a 5975C-7890A system (Agilent, USA) with a DB-5 capillary column (30 m × 0.25 mm, 0.25 μm film thickness). In addition, helium was used as the carrier gas at a constant flow rate of 1.0 mL min^− 1^. The column temperature program was set to 60 °C (2 min) and 60–260 °C (10 °C min^− 1^, hold time of 10 min). The injection and detection temperatures were maintained at 200 and 250 °C, respectively. Electron ionization (EI) mass spectra were acquired in full-scan mode from 50 to 55 m/z. The compounds were identified by comparing their mass spectral data to those in NIST library.

### Preparation of RNA for transcriptome analysis

The four samples of symbiotic seed germination were collected for transcriptome analysis. Additionally, samples of the pure cultured *C. disseminatus* on OMA medium were collected for RNA de novo assembly. Total RNA was extracted from 100 mg using plant RNeasy Kit (Omega Bio-Tek, USA) according to the manufacture’s protocol. The extracted RNA was treated with RNase-free DNase I to remove genomic DNA. The quality and quantity of the purified RNA were tested by using NanoDrop One (Nanodrop Technologies Inc., CA, USA), Agilent 2100 Bioanalyzer (Agilent Technologies, Santa Clara, CA, U.S.A.) and 1% agarose gel electrophoresis. Library construction and sequencing were performed using Illumina HiSeq4000 platform (Illumina Inc., CA, USA) at Beijing Genomics Institute (Shenzhen, China).

### De novo assembly of RNA-seq data and functional annotation of unigenes

The total clean reads were obtained by removing reads containing adapters, ambiguous reads (*N* > 5%), and low-quality reads from the raw reads. De novo assembly of the trimmed reads derived from each sample was conducted using Trinity V2.0.6 with default settings. The complete series of fungal and plant transcriptomic data were deposited in the National Center for Biotechnology information (NCBI) database (accession: No. PRJNA762946).

Unfortunately, reference genomes of *C. appendiculata and C. disseminatus* have not been published yet. We were unable to remove the intracellular hyphae from the symbiotic protocorms. Therefore, the fungus *C. disseminatus* de novo group was assembled individually using Trinity at default parameters. The coding sequences of symbiotic protocorms were first mapped to the fungal de novo assembly nonredundant protein (NR) database. Then, all the mapped *C. disseminatus* reads were derived from the symbiotic group [15, 47]. The remaining unpaired reads were classified as the de novo reference assembly of *C. appendiculata* and were prepared to detect the expression levels. The paired-end reads were mapped to each plant and fungal reference genome assembly using Bowtie2 V2.2.5 [19]. The transcript abundance was estimated using RSEM V1.2.28 [21]. The gene expression levels were estimated by applying the fragments per kilobase per million mapped reads (FPKM). Functional annotation of the unigenes was subjected to NR, Nt, KOG, GO, KEGG, Swissprot and Pfam public databases.

### Expression analysis of DEGs

The differentially expressed genes (DEGs) in library size were identified with DESeq2 and moderated estimation of the fold change [1, 25] in the expression level with a fold change ≥2.0 and Qvalue ≤0.05. The GO and KEGG enrichment were performed using the obtained DEGs.

### Statistical analysis

All experiments were repeated at least three times and the results were presented as means ± SD. The statistical analysis was performed using one-way repeated measures ANOVA, and significant differences were tested by Duncan’s test at *p* < 0.05.

## Results

### Lignocellulose degradation and water uptake of *C. appendiculata* seeds during symbiotic germination

**Fig. 2:**
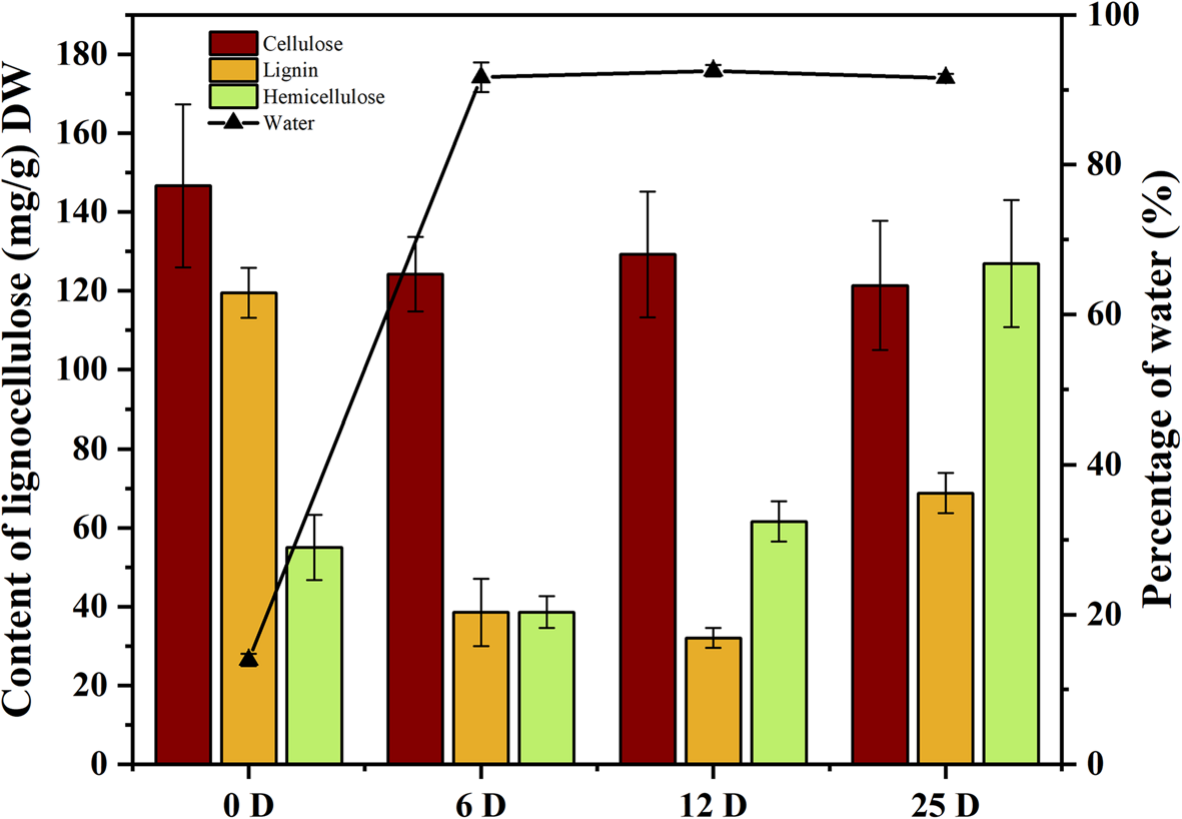
Lignocellulose content and percentage of water in *C. appendiculata* seeds during symbiosis. CA, SY1, SY2 and SY3 indicated symbiotic germination at 0 day, 6 days, 12 days, and 25 days after sowing, respectively

To investigate the degradation of lignocellulose by *C. disseminatus*, the loss of lignocellulose dry weight and water absorption during symbiosis were measured. After 6 days of symbiotic germination of *C. appendiculata* with *C. disseminatus*, the lignin content significantly dropped from 119.48 mg g^− 1^ (CA) to 38.55 mg g^− 1^ (SY1) and the lignin degradation rate in the seeds was 73.15% at 12 days (SY2) (Fig. 2). Subsequently, with cell differentiation and protocorm development, lignin accumulation increased to 68.84 mg g^− 1^ at SY3, but remain much lower than that of CA. The hemicellulose content was also decreased from 55.04 mg g^− 1^ in CA to 38.65 mg g^− 1^ in SY1. The hemicellulose degradation rate was 29.78% in SY1, and significantly increased to 61.61 mg g^− 1^ in SY2. With protocorm formation, the hemicellulose content increased to 126.89 mg g^− 1^ in SY3. The cellulose content did not change significantly among different stages.

Under symbiosis, the water content increased sharply significantly from CA to SY1 (13.94 and 91.66%, respectively) and then unchanged at SY2 and SY3 (92.54 and 91.58%, respectively) (Fig. 2). These results revealed that the lignified seed coat was broken by *C. disseminatus*, resulting in enhanced water permeation to the embryo. The results also demonstrate that the seed coat limitation is one of the important components of seed dormancy.

### Identification of lignocellulose in symbiotic seeds

**Fig. 3:**
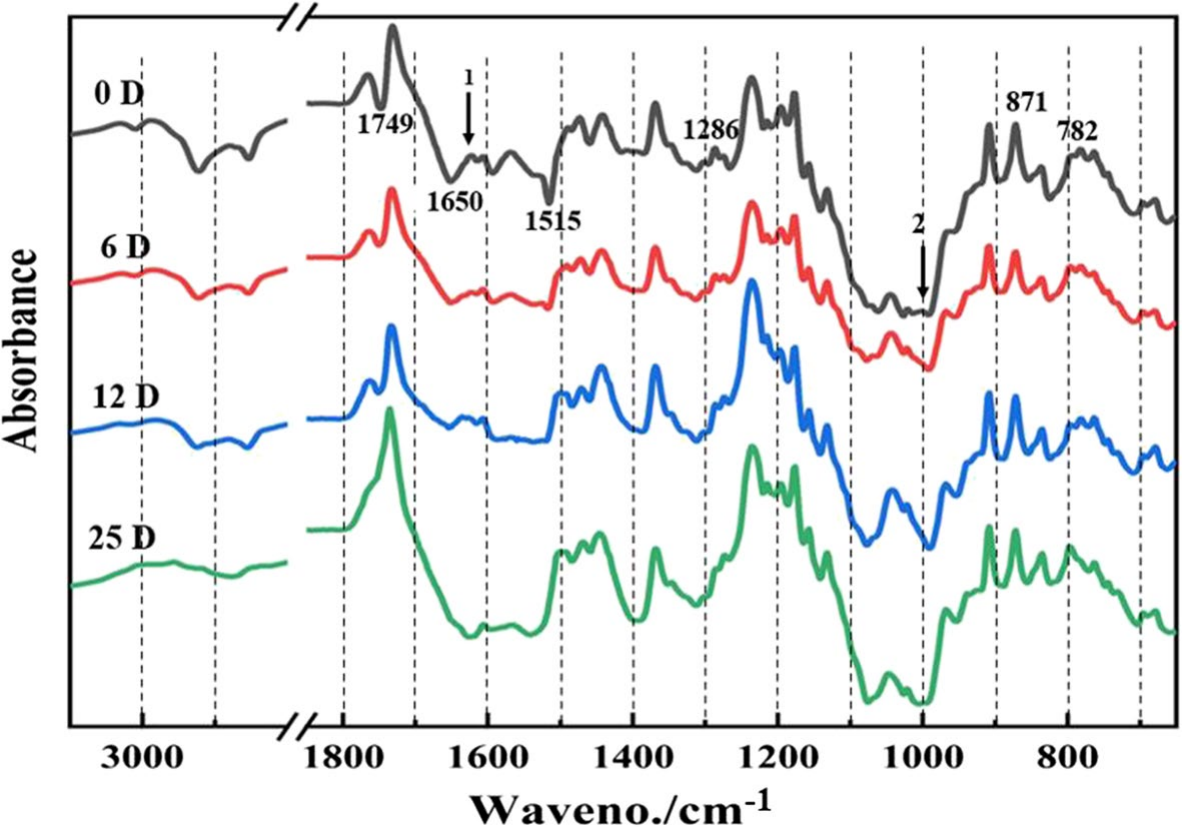
FTIR spectra of *C. appendiculata* seeds at different stages of symbiotic germination with *C. disseminatus*. CA, SY1, SY2 and SY3 indicated symbiotic germination at 0 day, 6 days, 12 days and 25 days after sowing, respectively

To further validate the lignocellulose degradation in *C. appendiculata* seeds by *C. disseminatus*, the chemical compositions of different samples was characterized by FTIR and Py-GC/MS. The samples showed remarkably different spectra (Fig. 3). Bands of C-lignin in CA were evident at 1650 (strong) and 782 cm^− 1^. Bands of G/S lignin existed at valleys of 1592, 1515 (sharp) and 871 cm^− 1^ (sharp). Under symbiosis with *C. disseminatus*, significant reduction in the lignin signals were recorded at 1650, 1592 and 1515 cm^− 1^ in SY1, SY2 and SY3, respectively as compared to CA. The xylan band of 2923 cm^− 1^ was significantly decreased at SY1, SY2 and SY3. The absorption band at 1621 cm^− 1^ (arrow 1) may be associated with carbonyl and acetyl groups in xylan degradation intermediates. The band, which was associated with cellulose, disappeared at 1000 cm^− 1^ (arrow 2) during symbiosis. In addition, strong and sharp signals were recorded at 1766 and 1749 cm^− 1^, though it was not clear whether or not they were associated with lignocellulose.

Pyrolysis of the seed symbiotic samples released various families of compounds (Table 1). The fraction of phenolic compounds decreased significantly, while the concentrations furanics and other compounds varied in different samples. These results suggested that the lignocellulose fraction of the seeds was degraded by the symbiotic fungus. In particular, lignin degradation was more significant as demonstrated. Other non-phenolic compounds such as acetic acid and ketone groups resulted from lignin and from the efficient release of phenolic monomers.

**Table 1:** Part of the chemical compounds released after Py-GC/MS of *C. appendiculata* seeds and seedings of CA, SY1, SY2 and SY3

Compound name	MW	Molecular formula	Retention time (min)	CA	SY1	SY2	SY3
Toluene	92	C_7_H_8_	3.472	+	+	–	–
Cyclobutanol	72	C_4_H_8_O	4.247	+	–	–	–
1-Cyclohexylethylamine	127	C_8_H_17_N	4.422	+	–	–	–
Carbon dioxide	44	CO_2_	4.503	+	–	–	–
Phenol	94	C_6_H_6_O	6.060	–	+	+	+
2-Acetyl-5-methylfuran	124	C_7_H_8_O_2_	7.348	+	–	–	–
4-Ethyl-phenol	122	C_8_H_10_O	8.001	+	+	+	–
Isovaleric acid, 3-ethylphenyl ester	206	C_13_H_18_O_2_	8.141	+	–	–	–
2-Methoxy-5-methylphenol	138	C_8_H_10_O_2_	8.257	+	–	+	–
4-Ethyl-2-methoxy-phenol	152	C_9_H_12_O_2_	9.114	+	–	–	–
2-Methoxy-4-vinylphenol	150	C_9_H_10_O_2_	9.458	+	+	+	–
Vanillin	152	C_8_H_8_O_3_	10.361	+	+	+	–
Vanillic acid	168	C_8_H_8_O_4_	10.641	+	–	–	–
Trans-isoeugenol	164	C_10_H_12_O_2_	10.693	+	+	+	–
1-(3,4-Dimethoxyphenyl)-ethanone	180	C_10_H_12_O_3_	11.644	+	–	–	–

### GC/MS analysis of low-molecular-weight products of degradation of lignocellulose of *C. appendiculata* seeds symbiotically germinated with *C. disseminatus*

To identify the products of lignocellulose biodegradation, GC-MS was used to detect the low-molecular-weight compounds in OMA symbiotic medium collected at different stages during the symbiotic germination of *C. appendiculata* seeds with *C. disseminatus* (Table 2; Fig. S1). The degradation products are listed in Table 2. More than 10 degradation products were detected. However, the CA group contained only dibutyl phthalate, suggesting that the compound might have originated from the OMA medium.

**Table 2:** The chemical composition of lignocellulose degradation products analyzed by GC-MS

Retention time (min)	Identified compound	CA	SY1	SY2	SY3
8.253	Benzeneethanamine, N-butyl-β,4-bis(trimethylsloxy)-	–	+	+	+
9.693	Dimethyl adipate	–	–	–	+
10.746	Benzene, 1,2,3-tris((trimethylsilyl) oxy)-	–	–	+	+
12.980	Benzeneacetic acid, 4-(trimethylsilyloxy) -3-methoxy	–	–	+	–
12.987	Ethanedioic acid	–	+	–	–
14.983	Phosphoric acid 2-(methoxyimino) ethylbis (trimethylsilyl) ester	–	+	–	–
16.704	Acetic acid, o-(trimethylsoloxy) phenyl-trimethylsily ester	–	+	+	+
18.213	Dibutyl phthalate	+	+	+	+
19.652	Mandelic acid, di(tert-butyldimethylsilyl)-	–	+	–	+
20.911	Mercaptoacetic acid, bis (trimethylsilyl)-	–	+	+	–
22.105	Butanoic acid, 2-((trimethylsilyl)oxy-)-, trimethylisly ester	–	–	+	–
22.126	Benzamide, N-benzyl-N-ethyl-ρ-isopropyl	–	+	–	–
22.648	Cis-9-oxabicyclo (6.1.0) no name	–	–	+	–
23.388	Di (2-ethylhexyl) phthalate	–	+	–	+
25.079	1, 2-BenzenediCArboxylic acid, bis (trimethylsilyl) ester	–	+	–	–

It is well documented that benzene compounds result from degradation of lignin. Five relative compounds (benzeneethanamine, benzene, benzeneacetic acid, benzamide and benzenedicarboxylic acid) were identified in the OMA symbiotic medium of SY1 and SY2. This suggested that the lignin component of the seed coat was degraded, in agreement with FTIR and Py-GC/MS data. Furfural, acetic acid and butanoic acid are degradation products of xylan. Acetic acid was detected in SY1, SY2 and SY3, whereas butanoic acid was detected only in SY2. Furfural was not detected at any stage. Some other acids and eaters resulting from lignocellulose degradation were detected in SY1 or SY2. These results confirmed that the lignocellulose fraction of the seed coat was degraded by *C. disseminatus* during symbiotic germination.

### RNAseq analysis, de novo assembly, and functional annotation

Analysis of RNA-seq of *C. appendiculata* at four developmental stages of symbiotic germination was performed to characterize the transcriptome changes during seed germination. A total of 41.06 Gb clean data were generated from each library after filtering out the low-quality data. Among raw reads in all samples, the Q30 values ranged from 91.47 to 92.28% indicating high-quality reads appropriate for further analysis (Table S1). Since no reference genome was available for *C. appendiculata*, all 359,510,000 reads were do novo-assembled into 48,750 CDS with an N50 length of 1398 bp and 97,800 unigenes with an average length of 1120 bp (Table S2). Samples of different biological replicates were clustered separately based on their distinct developmental stages.

**Fig. 4:**
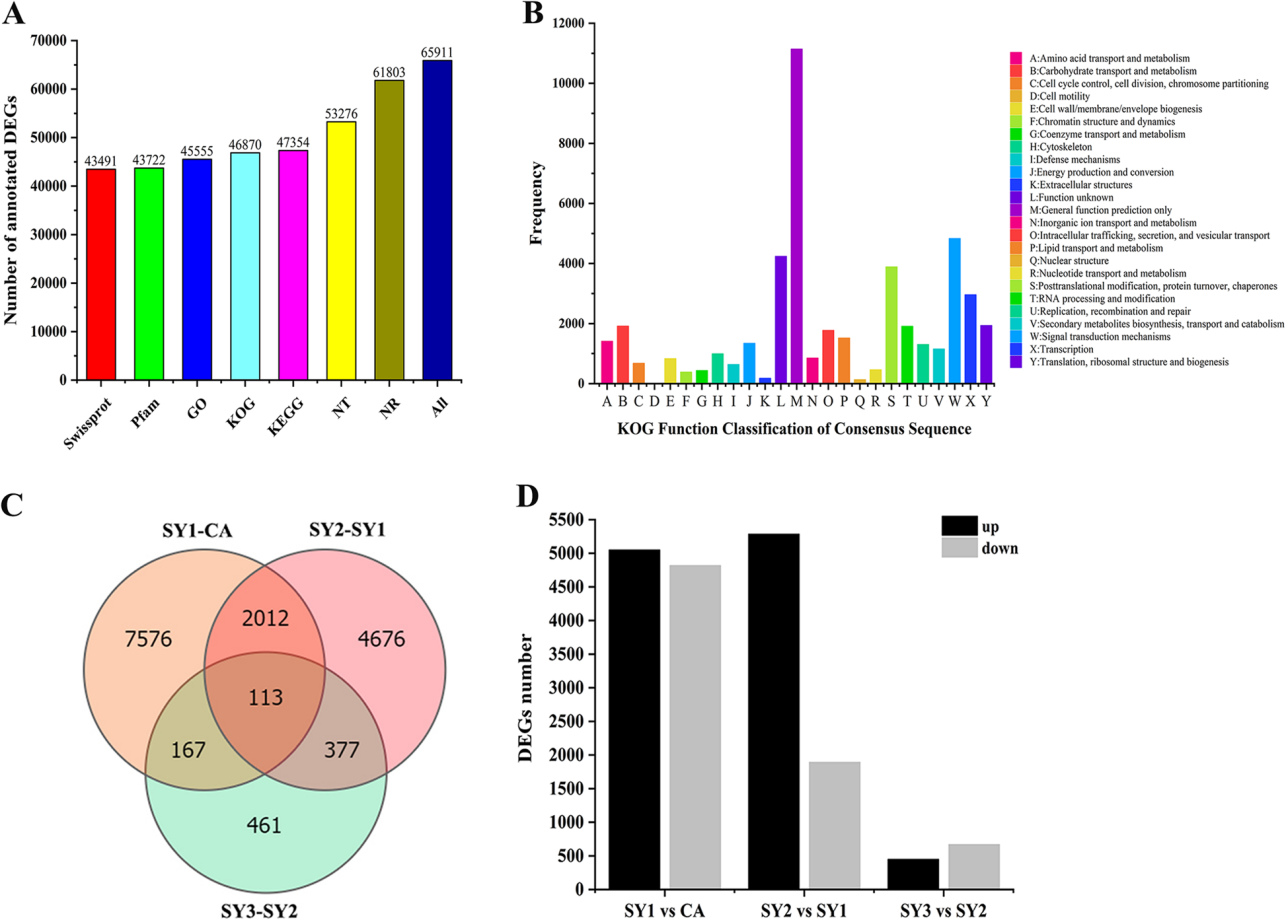
Statistical analysis of the annotated and differetially expressed unigenes (DEGs) during *C. appendiculata* symbiotic seed germination. **A.** Numbers of unigenes annotated into Swissprot, Pfam, GO, KOG, KEGG, NT, NR and total number of annotated unigenes. **B.** Functional classification of unigenes annotated into KOG. Capital letters A-Y represent the functional categories. **C.** Venn diagram of all DEGs. **D.** Up/down-regulated unigenes in all development stages. The total number of DEGs peaked between SY1 and CA. CA, SY1, SY2 and SY3 indicated the symbiotic seeds at 0 day, 6 days, 12 days, and 25 days after sowing,respectively

For annotation, 97,800 unigenes were subjected to BLASTX search against the sequences in NR, Nt, Pfam, Swissprot, GO, KOG, and KEGG databases (Fig. 4A; Table S3). As a result, a total of 65,911 unigenes (67.39% of all unigenes) had at least one putative function from one of these databases. For NR annotation, a total of 61,803 (63.19%) unigenes were annotated. As shown in Fig. S2, 36,107 unigenes were assigned with a best score to *Dendrobium catenatum* (58.42%) and 14,862 ones were assigned to *Phalaenopsis equestris* (24.05%). For GO annotation, 45,555 unigenes were annotated into three GO categories, including cellular component (CC), molecular function (MF), and biological process (BP) (Fig. S3). The cellular component class contained cellular anatomical entity and intracellular and protein-containing complex. The most abundant molecular functions were binding and catalytic activity. Among the biological processes, cellular and metabolic processes were more abundant. Other entries such as biological regulation, localization and response to stimulus were relatively high. Unigenes among different groups were chiefly classified into carbohydrate transport and metabolism, function unknown, general function prediction only, posttranslational modification, protein turnover, chaperones, signal transduction mechanisms and transcription (Fig. 4B).

### Functional annotation of differentially expressed genes

#### Differential expression analysis of gene expression at different stages of symbiotic germination

To identify the genes involved in symbiosis, we compared, as pairs, the transcriptomes of the four developmental periods of the symbiotic seeds. The pair SY1-CA, represented the symbiotic seeds at 6 days (fungal invasion) compared to 0 day (fungus-free seeds), while SY2-SY1 was the symbiotic material at 12 days (fungal colonization) compared to the stage of fungal invasion at day 6. The pair SY3-SY2 presented the transition to fungal degradation and protocorms with meristem from the fungal colonization at 12 days. A total of 15,382 genes showed significantly different expression levels (log2 FPKM≥2.0, Qvalue≤0.05) among all libraries (Fig. 4C). Among these differentially expressed genes (DEG), 5050 genes were up-regulated and 4818 ones were down-regulated in SY1-CA (Fig. 4D). In SY2-SY1, 5285 genes were up-regulated and 1893 ones were down-regulated in SY2-SY1. In SY3-SY2, 449 genes were up-regulated and 669 genes were down-regulated (Fig. 4D; Table S4). We found that the highest number of DEGs was shared between the early mycelium penetration stages (SY1-CA), followed by the early germination stage (SY2-SY1), and then the protocorm stage (SY3-SY2) (Fig. 4D).

#### GO enrichment analysis

To characterize the DEGs, we performed a GO enrichment analysis using Rage tool. Among the GO terms of DEGs, there were 51, 100 and 153 terms significantly overrepresented in SY1-CA, SY2-SY1 and SY3-SY2, respectively (Table S5). In total, 25 GO terms were over-represented in the three comparisons (Table 3). In particular, these terms included some significantly overrepresented GO terms related to oxidation reactions such as heme binding, monooxygenase activity, dioxygenase activity, peroxidase activity and iron ion binding. In addition, the terms of response to water, metal iron binding and endo-1,4-β-xylanase activity were annotated in SY1 versus CA. The terms of ATP binding, channel activity and cell wall macromolecule catabolic process were identified in SY2-SY1. However, xyloglucan and carbohydrate metabolic process were also found.

**Table 3:** Significantly overrepresented gene ontology terms during the symbiotic germination of *C. appendiculata* with *C. disseminatus* DJF-10

Code	GO Term	SY1/CA	Q-value SY2/SY1	SY3/SY2
**Molecular function**				
● GO:0020037	Heme binding	1.53E-11	1.17E-09	2.19E-14
● GO:0004497	Monooxygenase activity	1.92E-11	8.15E-09	9.85E-12
GO:0051213	Dioxygenase activity	7.88E-05	9.08E-05	0.000427
GO:0004601	Peroxidase activity	0.000302	0.028842	2.37E-05
● GO:0016758	Transferase activity, transferring hexosyl groups	0.000308	2.82E-06	0.000111
● GO:0016705	Oxidoreductase activity, acting on paired donors, with incorporation or reduction of molecular oxygen	0.000955	0.002064	5.36E-06
● GO:0005506	Iron ion binding	0.00119	0.034884	2.71E-05
GO:0051787	Misfolded protein binding	0.002192	0.001156	2.22E-07
GO:0016747	Transferase activity, transferring acyl groups other than amino-acyl groups	0.002288	0.003067	4.85E-11
GO:0005200	Structural constituent of cytoskeleton	0.002973	0.012555	0.000248
GO:0003700	DNA-binding transcription factor activity	0.006013	0.005378	2.42E-06
GO:0031072	Heat shock protein binding	0.005378	6.13E-05	6.74E-15
GO:0051082	Unfolded protein binding	0.029129	9.41E-11	6.95E-38
GO:0030246	Carbohydrate binding	0.046851	2.05E-21	5.90E-08
**Cellular component**				
GO:0048046	Apoplast	3.80E-05	8.62E-05	2.74E-09
GO:0005576	Extracellular region	0.020282	2.24E-06	8.25E-14
**Biological process**				
GO:0042744	Hydrogen peroxide catabolic process	3.73E-06	0.001527	1.08E-06
GO:0006979	Response to oxidative stress	0.000395	0.007245	5.89E-10
GO:0006986	Response to unfolded protein	0.000395	0.007245	5.89E-10
GO:0034620	Cellular response to unfolded protein	0.001175	0.01359	1.84E-09
GO:0007017	Microtubule-based process	0.001722	0.005323	0.000165
GO:0051085	Chaperone cofactor-dependent protein refolding	0.002231	0.003581	3.08E-10
GO:0009664	Plant-type cell wall organization	0.006583	0.000291	0.000293
GO:0045490	Pectin catabolic process	0.007005	0.000102	0.013441
GO:0006457	Protein folding	0.047891	4.43E-09	2.36E-25

Round (filled) circles represent the GO terms were overrepresented during mycorrhizal fungus *Tulasnella* sp. symbioses with *Bletilla striata* [29]

#### KEGG enrichment analysis

To investigate the differences in metabolic processes at different stages of symbiotic germination in more detail, a KEGG pathway enrichment analysis with the DEGs was conducted, based on the Qvalue≤0.05 (Table S6, S7). At SY1-CA, the categories flavonoid biosynthesis, plant hormone signal transduction, fatty acid degradation and phenylpropanoid biosynthesis were significantly enriched. The pathways of phenylpropanoid and flavonoid biosynthesis were also dramatically enriched in SY2-SY1 and SY3-SY2. Pathways for fatty acid degradation, carbon fixation in photosynthetic organisms, glycosaminoglycan degradation and carbon metabolism were enriched in SY2-SY1. Pathways of protein processing in endoplasmic reticulum, glycosaminoglycan degradation, plant signal transduction and other glycan degradation were significantly enriched between SY3-SY2. Taken together, these findings suggested that the fungus induced the expression of plant hormone signal transduction pathways which may break the seed dormancy. Moreover, it induced the expression of fatty acid degradation, carbon metabolism and endoplasmic reticulum processing, and hence improves the efficiency of utilization of stored nutrients.

#### Expression patterns of symbiosis-related genes at each stage

**Fig. 5:**
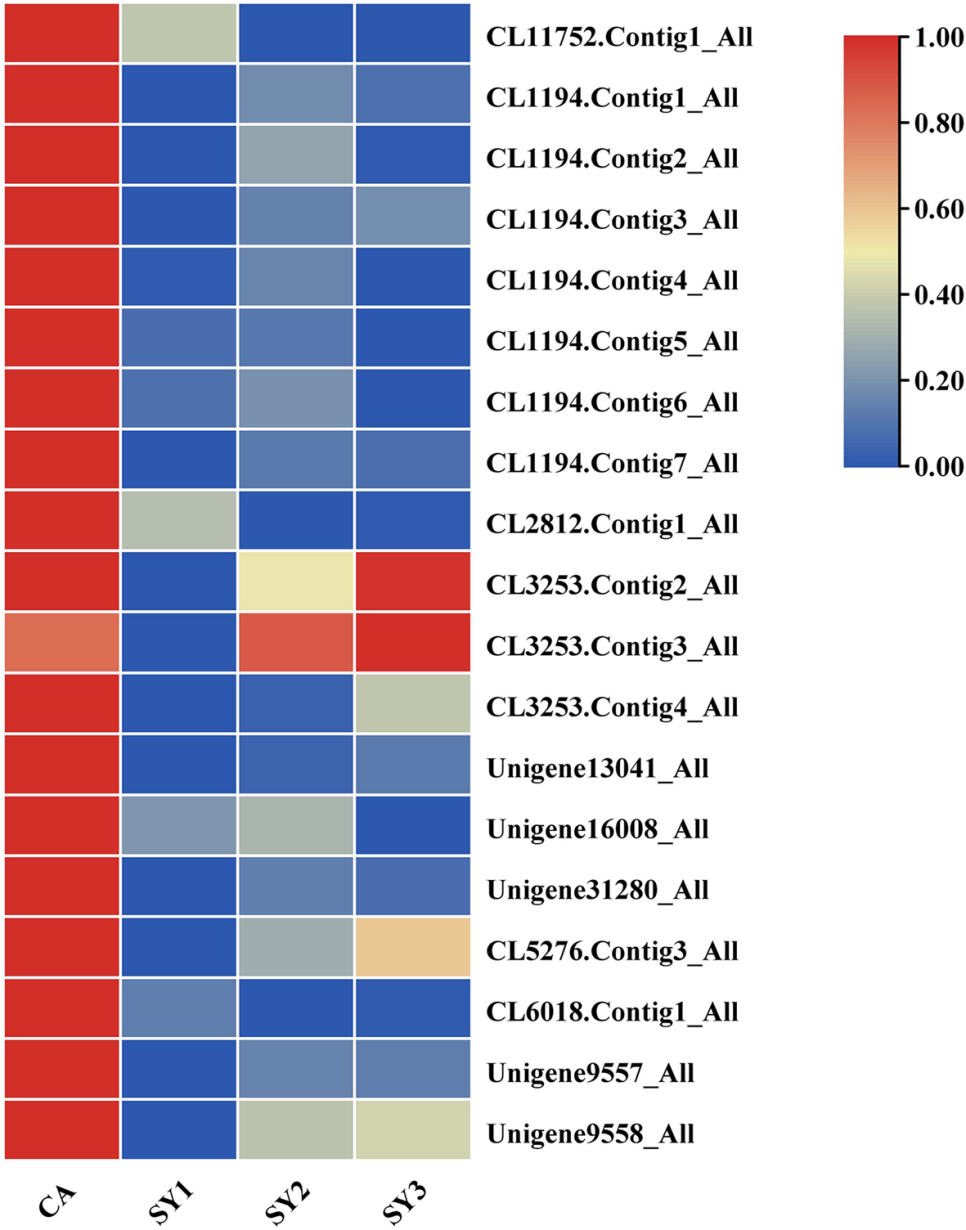
Cluster heatmap of response to water and ABA biosynthesis term-related gene expression in *C. appendiculata* during symbiotic germination. CA, SY1, SY2 and SY3 represented symbiotic seeds at 0 day, 6 days, 12 days and 25 days after sowing, respectively

The lignified seed coat forms a hydrophobic barrier to water permeation during germination. The lignin content of the seeds was sharply reduced after symbiosis with the fungus *C. disseminatus*. Once the hydrophobic barrier was removed, the embryo was able to absorb water efficiently. In this study, we paid much attention to terms related to water absorption. Fifteen genes (including 9 *CaDHN1* genes, 4 *CaXero1* genes, 1 hypothetical protein, and 1 *CaDHN4*-like) related to the term response to water were down-regulated (Fig. 5; Table S8), and remained at low expression levels in SY2 and SY3. Among these genes, the expression of five *CaDHN1* genes (CL1194.Contig2_All, CL1194.Contig3_All, CL1194.Contig5_All, Unigene16008_All and Unigene31280_All) decreased from 176.4, 241.7, 311.4, 166.1 and 38.2 in CA to 0.3, 0.6, 5.4, 4.1 and 0 in SY1, respectively. Two *CaXero1* genes (CL1194.Contig6_All and CL1194.Contig7_All) showed high expression levels of 504.1 and 600.1, respectively at CA, but were down-regulated to 4.1 and 12.0, respectively in SY1. The expression level of a hypothetical protein (CL1194.Contig1_All) decreased from 229.7 in CA to 2.9 in SY1. The results suggested that these genes regulate the response to fungal stimulation during seed germination of *C. appendiculata* after symbiosis with the mycorrhizal fungus.

Indeed, plant hormones play a vital role in the process of symbiotic germination. The current study revealed that a large number of DEGs was annotated to signal transduction mechanisms. Carotenoid biosynthesis provides the precursors for the synthesis of ABA. In our study, zeaxanthin epoxidase *ZEP* (CL5276.Contig3_All), 9-cis-epoxycarotenoid dioxygenase *NCED3* (CL6018.Contig1_All) and β-carotene hydroxylase (Unigene9557_All and Unigene9558_All) were significantly down-regulated in SY1-CA (Fig. 5; Table S8). Furthermore, most of the abscisic acid receptor pyrabactin resistance 1-like (*PYLs*) were up-regulated in SY1-CA. Seven genes of protein phosphatase 2C (*PP2C*), six of SNF1-related protein kinase 2 (*SnRK2*) and four basic region-leucine zipper (*bZIP*) transcription factors were down-regulated in this group (Fig. 5; Table S8).

## Discussion

The orchid *C. appendiculata* is one of the most difficult-to-propagate species, a condition which severely restricts its large-scale production. The factors that inhibit *C. appendiculata* seed germination are still largely unknown. During symbiosis, the lignin content abruptly decreased resulting in a significant increase in water uptake of the seeds compared to the control. These results revealed a negative correlation between lignin and germination, suggesting a possible mechanism whereby the testa-imposed dormancy was broken [34, 42]. The mycorrhizal fungus *C. disseminatus* can degrade lignocellulose by releasing various enzymes such as xylanase, laccase and cellulase [2, 39]. In the present study, we tested laccase, xylanase and cellulase in the symbiotic OMA medium during symbiosis. The results showed that these enzymes were abundant in SY1 and SY2 but decreased at SY3 (Fig. S4) consistently with the physiological data which indicated that lignocellulose was largely degraded at SY1 and SY2. However, the chemical basis of the mechanism involved has not been clearly explained. Moreover, a layer of the seed coat was strained greenish blue with TBO, indicating the presence of polyphenols and lignin in the seed coat of mature seeds, a barrier that disappeared during symbiotic germination [14]. Moreover, the enzymes of cellulases may play an important role in the penetration of fungal hyphae into orchid seeds [35], suggesting that the mycorrhizal fungus *C. disseminatus* essentially scarifies and degrades the lignified seed coat by producing hydrolytic enzymes, resulting in facilitating water uptake and stimulating germination.

Some microorganisms grow on lignocellulose as carbon source and degrade lignin to access cellulose as a preferred carbon source [11, 27]. It is noteworthy that *C. disseminatus* is a common species that exists on stumps, buried wood and logs with high wood-decaying capacity [26, 30, 39]. Hynson et al. [17] reported that more than 80% of the *C. appendiculata* carbon was obtained from fungal partner, which is more than that in many other orchids. In the present study, the reducing sugar concentration was highest at SY1 in the OMA medium, and decreased thereafter (Fig. S5). The mycelium formed pelotons in the embryo that began to degrade after about 2 weeks. Combing these data with these of enzyme activity, lignocellulose analysis and germination experiment, it seems that *C. appendiculata* was associated with the wood-decaying *Coprinellus* members [14, 45] that obtains nutrients via saprotrophic fungi for seed germination and protocorm development during symbiosis [36, 43]. Fungal invasion appears to trigger metabolic changes in the host plant (especially the nutrient accumulation) during the establishment of symbiosis between *C. appendiculata* and *C. disseminatus*, a process that requires further research.

Four key stages were selected for transcriptome analysis based on the dynamics of fungus penetration into the embryo and key anatomical features in the embryo development. Unfortunately, it was not possible to remove the intracellular fungal hyphae from the symbiotic samples. This meant that the symbiotic samples contained transcripts of *C. appendiculata* and the fungus *C. disseminatus*. Therefore, RNA from the symbiotic fungus library was used for establishing a *C. disseminatus* reference transcriptome and all the mapped reads were driven from the symbiotic samples. This method could be effectively applied to assign genes identified in the transcriptome of plant-fungi associations [47]. The orchid *C. appendiculata* has been reported to form mycorrhizal associations with saprotrophic fungi [14, 43, 45], which have the potential to elicit strong defense responses in plants. However, our transcriptome data provided no evidence of strong defense activation at the GO terms in *C. appendiculata* symbiotic samples. This result is similar to those of other symbiotic transcriptome studies on orchids, such as *B. strilata* [29], *Serapias vomeracea* [33] and to arbuscular mycorrhizal AM symbiosis. These results suggested that plants recruit a signaling pathway to exploit saprotrophic fungi without inducing stress response [29].

The pathway of plant hormone signal transduction was highly enriched in SY1-CA during *C. appendiculata* symbiotic germination. The data showed that ABA content significantly dropped from 1.38 mg g^− 1^ (CA) to 0.08 mg g^− 1^ (SY2) with more than 15-fold reduction, and remained at low level thereafter. During this stage, the genes for key enzymes of ABA synthesis were down-expression. Meanwhile, the genes of *DHN* family were significantly enriched in SY1-CA, suggesting high expression in CA that effectively alleviate the negative effects of drought stress [37]. During symbiosis, the expression of *DHN* family was down-regulated. Therefore, we suggest that the low permeability of the seed coat and the high level of ABA in the embryo could act in parallel to hinder the germination of the mature seeds of *C. appendiculata*.

## Conclusion

In the present study, we investigated the biochemical and transcriptomic changes of *C. appendiuclata* seeds and provided an outline of the important components of the response to symbioses. In summary, the presence of lignified seed coats on the seeds of *C. appendiculta* suggests testa-imposed dormancy that restricts seed germination. As the lignified seed coat was broken down by mycorrhizal fungus *C. disseminatus*, the water absorption by the embryo was improved. The maximal reduction in lignin content was 67.34% at the first week after sowing. This result was confirmed by FTIR and Py-GC/MS of lignin degradation. Comparative transcriptome profiling revealed the expression patterns of water uptake and ABA biosynthesis candidate genes, which demonstrated that the testa-imposed dormancy was broken. During symbiosis, all genes related to water uptake and ABA biosynthesis were down-regulated. This study extends our understanding of the barriers to seeds germination in *C. appendiculata*. Future work should focus on the molecular mechanistic processes in *C. appendiculata* seed germination as affected by *C. disseminatus* symbiosis.

## Supplementary Information

**Additional file 1: Fig. S1.** GC-MS analysis of the chemical compositions of lignocellulose degradation products during symbiotic germination. **Fig. S2.** NR annotated species distribution of *Cremastra appendiculata*. *Dendrobium catenatum* shows the highest similarity. **Fig. S3.** GO function annotation. The most abundant functions are binding and catalytic activity in terms of molecular function and cellular anatomical entity in terms of cellular component. **Fig. S4.** Variation of the enzymes during symbioses. **Fig. S5.** Variation of the reducing sugar concentration produced in the OMA medium during symbioses.

**Additional file 2: Table S1.** Overview of transcriptome sequence and de novo assembly results. **Table S2.** Overview of transcriptome assembly showing length of CDS and unigenes. **Table S3.** Summary of functional annotation of contigs from BLAST searches against public databases. **Table S4.** DEGs richment (log_2_ FC ≥ 2, Qvalue≤0.05). **Table S5.** Significantly overrepresented gene ontology terms during the symbiotic germination of *C. appendiculata* with *C. disseminatus* DJF-10. **Table S6.** Significantly enriched KEGG pathway of different samples. **Table S7.** DEGs enrichment of KEGG during symbiotic germination. **Table S8.** DEGs related to water and ABA biosynthesis.

## Data Availability

The RNA-Seq data has been deposited in the Sequence Read Archive (SRA) at the National Center for Biotechnology Information (NCBI). The accession number is PRJNA762946, which includes 11 accession items (SRX12177800, SRX12177801, SRX12177802, SRX12177803, SRX12177804, SRX12177805, SRX12177806, SRX12177807, SRX12177808, SRX12177809, SRX12177810).

## Abbreviations

*C. appendiculata*: *Cremastra appendiculata*
*C. disseminatus* DJF-10: *Coprinellus disseminatus* DJF-10
OMA: Oatmeal agar medium
CA: *Cremastra appendiculata* symbiotic seeds at 0 day after sowing
SY1: *Cremastra appendiculata* symbiotic seeds at 6 days after sowing
SY2: *Cremastra appendiculata* symbiotic seeds at 12 days after sowing
SY3: *Cremastra appendiculata* symbiotic seeds at 25 days after sowing
Py-GC/MS: Pyrolysis-gas chromatography
FTIR: Fourier transform infrared spectroscopy
GC-MS: Gas Chromatography and Mass Spectrometry
DEG: Differentially expressed genes
NR: Non-redundant proteins
KEGG: Kyoto Encyclopedia of Genes and Genomes
GO: Gene Ontology
FPKM: Fragments per kilobase per million mapped reads
DHN: Dehydrin
ABA: Abscisic acid
ZEP: Zeaxanthin epoxidase
NCED3: 9-cis-epoxyCArotenoid dioxygenases
