# Supplementary figures and images for: Biochemical and transcriptomic analyses of the symbiotic interaction between Cremastra appendiculata and the mycorrhizal fungus Coprinellus disseminatus
Source: BMC Plant Biol. 2022 Jan 4;22:15. doi: 10.1186/s12870-021-03388-6 (PMC8725509; doi:10.1186/s12870-021-03388-6)

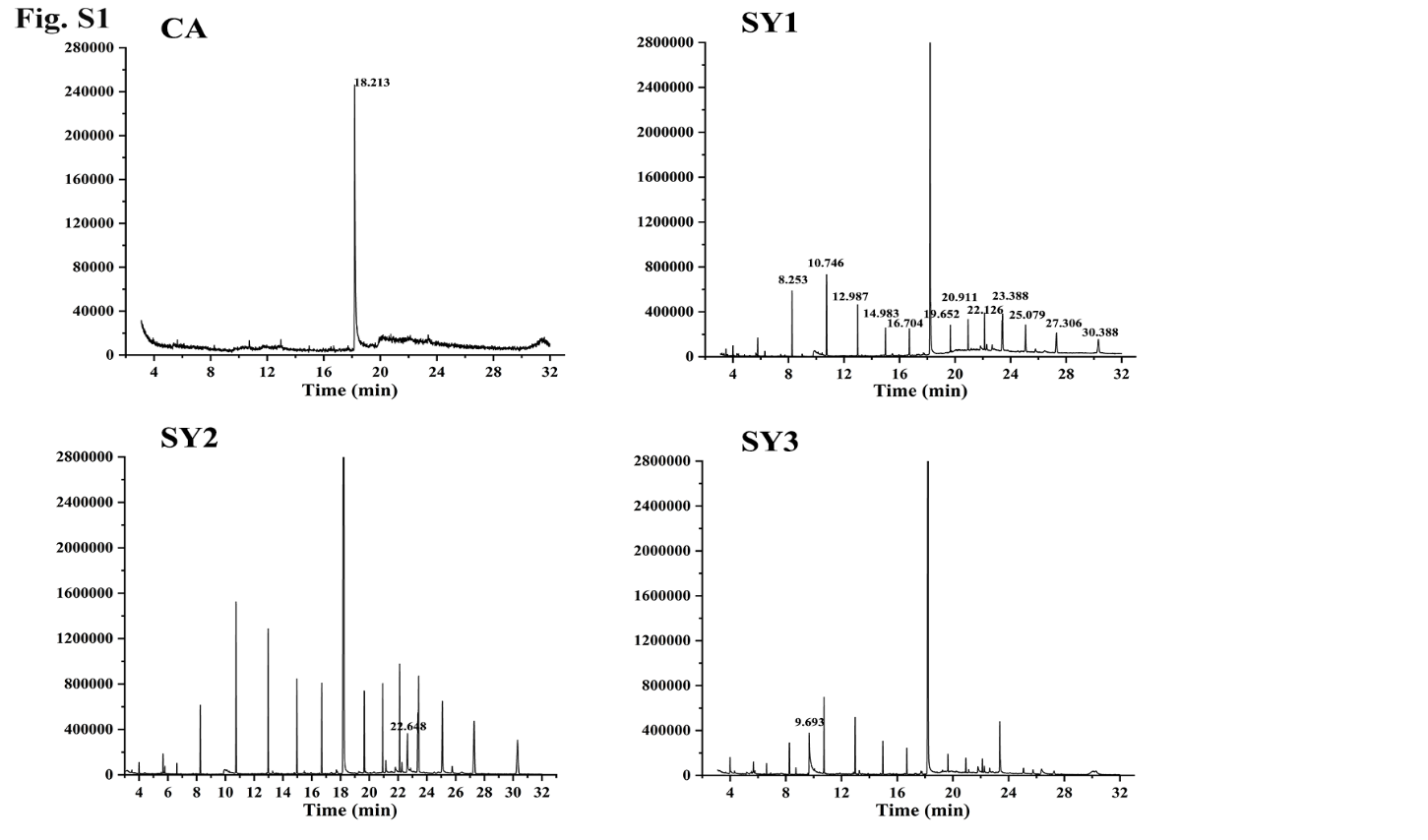


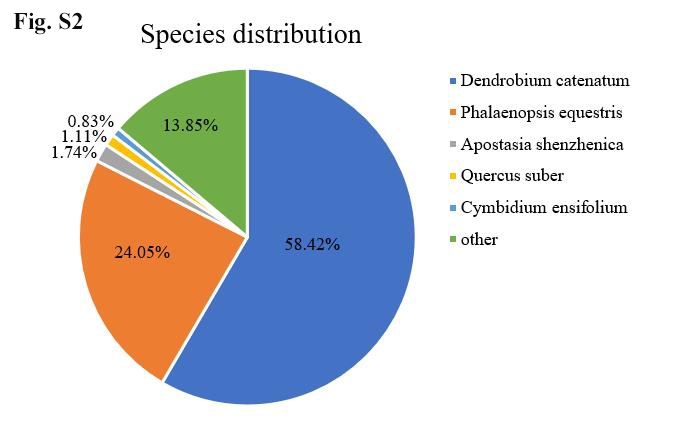


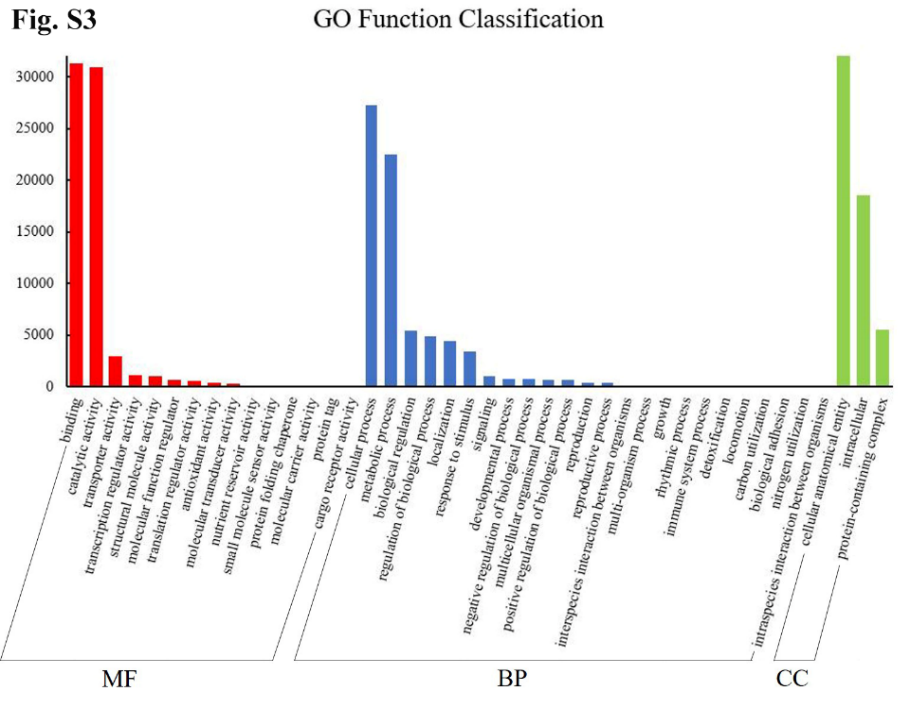


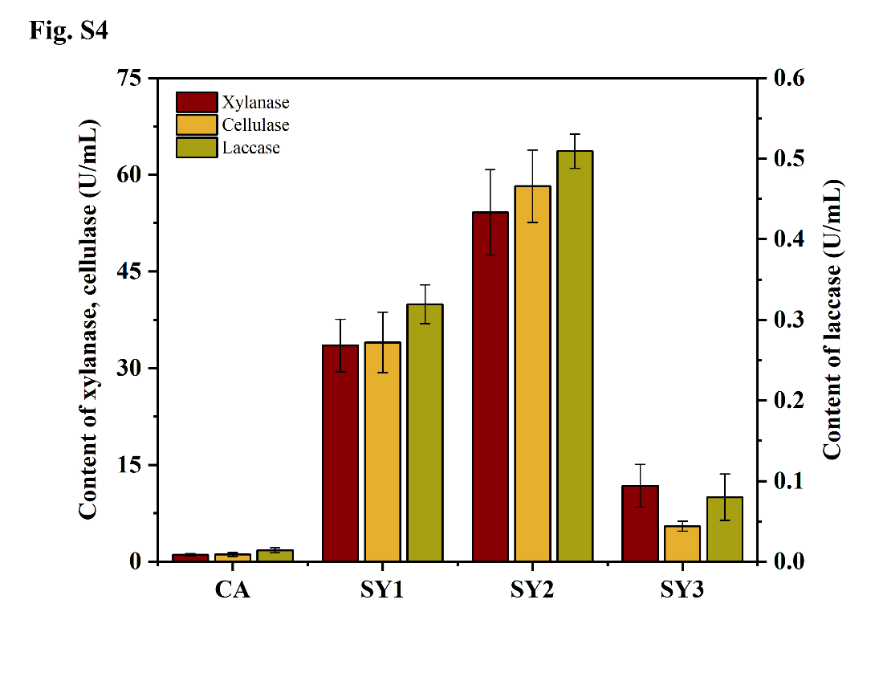


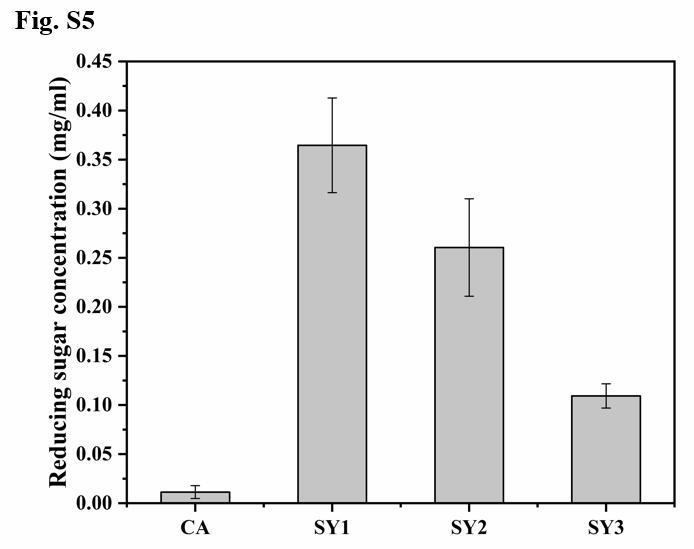

Supplement: Supplementary file 1 — Additional file 1: Fig. S1. GC-MS analysis of the chemical compositions of lignocellulose degradation products during symbiotic germination. Fig. S2. NR annotated species distribution of Cremastra appendiculata. Dendrobium catenatum shows the highest similarity. Fig. S3. GO function annotation. The most abundant functions are binding and catalytic activity in terms of molecular function and cellular anatomical entity in terms of cellular component. Fig. S4. Variation of the enzymes during symbioses. Fig. S5. Variation of the reducing sugar concentration produced in the OMA medium during symbioses. [file 12870_2021_3388_MOESM1_ESM.docx]
